# The need for national medical licensing examination in Saudi Arabia

**DOI:** 10.1186/1472-6920-8-53

**Published:** 2008-11-25

**Authors:** Sohail Bajammal, Rania Zaini, Wesam Abuznadah, Mohammad Al-Rukban, Syed Moyn Aly, Abdulaziz Boker, Abdulmohsen Al-Zalabani, Mohammad Al-Omran, Amro Al-Habib, Mona Al-Sheikh, Mohammad Al-Sultan, Nadia Fida, Khalid Alzahrani, Bashir Hamad, Mohammad Al Shehri, Khalid Bin Abdulrahman, Saleh Al-Damegh, Mansour M Al-Nozha, Tyrone Donnon

**Affiliations:** 1 Medical Education Unit, Faculty of Medicine, Umm Al Qura University, Makkah, Saudi Arabia; 2 National Guard Health Services, Jeddah, Saudi Arabia; 3 Faculty of Medicine, King Fahd Medical City, Riyadh, Saudi Arabia; 4 Medical Education Unit, Faculty of Medicine, Taif University, Taif, Saudi Arabia; 5 Faculty of Medicine, King Abdulaziz University, Jeddah, Saudi Arabia; 6 Faculty of Medicine, King Saud University, Riyadh, Saudi Arabia; 7 Medical Education Unit, Faculty of Medicine, King Faisal University, Dammam, Saudi Arabia; 8 Faculty of Medicine, King Saud Bin Abdulaziz University of Health Sciences, Riyadh, Saudi Arabia; 9 Medical Services Directorate, Ministry of Interior, Saudi Arabia; 10 Faculty of Medicine, King Khalid University, Abha, Saudi Arabia; 11 Faculty of Medicine, Al-Imam Mohammad Bin Saud University, Riyadh, Saudi Arabia; 12 Faculty of Medicine, Qaseem University, Saudi Arabia; 13 Faculty of Medicine, Taibah University, Madinah Munawwarah, Saudi Arabia; 14 Medical Education Research Unit, Faculty of Medicine, University of Calgary, Canada

## Abstract

**Background:**

Medical education in Saudi Arabia is facing multiple challenges, including the rapid increase in the number of medical schools over a short period of time, the influx of foreign medical graduates to work in Saudi Arabia, the award of scholarships to hundreds of students to study medicine in various countries, and the absence of published national guidelines for minimal acceptable competencies of a medical graduate.

**Discussion:**

We are arguing for the need for a Saudi national medical licensing examination that consists of two parts: Part I (Written) which tests the basic science and clinical knowledge and Part II (Objective Structured Clinical Examination) which tests the clinical skills and attitudes. We propose this examination to be mandated as a licensure requirement for practicing medicine in Saudi Arabia.

**Conclusion:**

The driving and hindering forces as well as the strengths and weaknesses of implementing the licensing examination are discussed in details in this debate.

## Background

**Figure 1:**
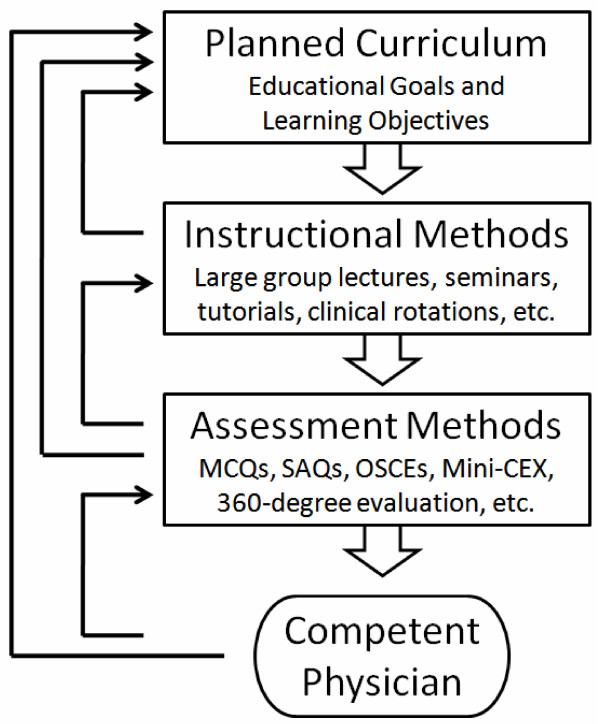
Dynamic Domains of Medical Education.

The three main interconnected domains of medical education are: curriculum design, instructional methods and assessment measures. Once the educational goals and learning objectives are stated within a curriculum, the instructional methods are selected to assure that the learning objectives are met, and then assessment measures are developed to ensure that the students have learnt what has been taught. Furthermore, educators and administrators are responsible to oversee the educational program to evaluate the need for changes to the learning objectives, instructional methods or assessment measures used. This cycle of ongoing program evaluation is based on the outcomes derived from the assessment measures selected (Figure 1). Of the three domains, the assessment component in medical education has quickly expanded with the introduction and refinement of new strategies and methods over the past three decades.

The current proposal is an initiative from a group of medical and educational professionals who are interested in enhancing the standards of medical education for the best of medical practice in Saudi Arabia. In this article, we will discuss the need for a national medical licensing examination in Saudi Arabia, its driving and hindering forces, along with the strengths and weaknesses of such an examination. The fact that Saudi Arabia does not have a national medical licensing examination is shared by many countries across the globe, and hence this debate can be extrapolated to other countries considering this licensure process as a mandatory requirement to practice medicine.

We anticipate opposing debates to our proposal, as it was the case with similar proposals for a change before. For example, the criticism against the addition of Part II of the Medical Council of Canada Qualifying Examination for Canadian graduates [1-5], the criticism against the addition of the Clinical Skills component to Step 2 of the United States Medical Licensing Examination by the National Board of Medical Examiners for graduates of medical schools in the United States (US) [6-8], the debate triggered by a proposal to establish a national licensing examination in Australia [9-11] and a similar debate by a consultation conducted by the General Medical Council of the United Kingdom (UK) to enquire about the current status of assessment of medical schools in the UK [12-15].

In this paper, we are not presenting new empirical evidence. In fact, we are summarizing the available debates on the need of a national licensing medical examination.

## Discussion

### The current status of medical education in Saudi Arabia

The first medical school in Saudi Arabia was established in 1967 at King Saud University. This was followed by the establishment of four medical schools over the span of thirty years (1967 to 1996). Since the beginning of the new millennium, 20 medical schools have been established (14) or planned for opening (6) in the coming few years. This will bring the total number of medical schools in Saudi Arabia to 25 (Table 1). The expansion in the number of medical schools is intended to meet the shortage of Saudi-national physicians, estimated to be less than 17% of the total physicians in 2000 [16] and to serve its population of around 28 million people and millions of tourists and pilgrims who visit Saudi Arabia annually.

**Table 1:** List of Medical Schools in Saudi Arabia

**Name**	**Location**	**Establishment Date**	**Sector**
King Saud University	Riyadh	1967	Government

King Abdulaziz University	Jeddah	1975	Government

King Faisal University	Dammam	1975	Government

King Khalid University	Abha	1982	Government

Umm Al Qura University	Makkah	1996	Government

Al Qassim University	Qassim	2000	Government

Taibah University	Madinah	2001	Government

King Faisal University	Al Ahssa	2001	Government

King Fahd Medical City	Riyadh	2004	Government

Ibn Seena	Jeddah	2004	Private

Jazan University	Jazan	2005	Government

King Saud Bin Abdulaziz University for Health Sciences	Riyadh	2005	Government

Taif University	Taif	2006	Government

The Batterjee College for Medical Sciences and Technology	Jeddah	2006	Private

Al Jouf University	Al Jouf	2007	Government

Najran University	Najran	2007	Government

Tabuk University	Tabuk	2007	Government

Alfaisal University	Riyadh	2008	Private

Al-Imam Mohammed Bin Saud Islamic University	Riyadh	2008	Government

King Saud University	Al-Kharj	2009	Government

Al-Marifa College of Medicine	Riyadh	2009	Private

Al-Rajhi College of Medicine	Qassim	2009	Private

Hail University	Hail	2009	Government

King Saud Bin Abdulaziz University for Health Sciences	Jeddah	2009	Government

King Saud Bin Abdulaziz University for Health Sciences	Al Ahssa	2009	Government

Twenty of the 25 medical schools are government-funded with no tuition fee for Saudi students. The five private medical schools are open to students of all nationalities; however, the average annual tuition fee is around $15,000 US. Mirroring the British system, all of the medical schools admit students with a high school diploma to enroll in a six year program. Some schools (e.g. King Saud bin Abdulaziz University for Health Sciences, established in 2005) offer an additional graduate students-stream of a four-year program with a requirement of a pre-medical Bachelor degree, mirroring the North American system. Acceptance into medical schools is based on students' high school grade point average, and performance on the General Aptitude Exam and National Achievement Test for Health Colleges. The latter two examinations, introduced four years ago, are national standardized tests conducted by the National Center for Assessment in Higher Education [17]. In addition, most schools include personal interviews in the selection process.

Each medical school in Saudi Arabia decides internally, by virtue of its curriculum committee, on the details of the curriculum and the educational objectives. Similarly, each medical school independently determines the instructional methods to be used to deliver the curriculum. The spectrum of educational strategies ranges from a lecture-based/teacher-centered to problem-based/student-centered approach to teaching and learning [18,19]. Finally, each medical school in Saudi Arabia develops and administers its own formative and summative assessment measures. Consequently, the methods of assessing students' knowledge, skills and attitudes in these medical schools are quite variable from one school to another. The written methods of assessment include multiple choice questions (MCQs), true-false questions, short answer questions, essays and modified essay questions. The oral methods of assessment include oral examination/viva, observed and unobserved long and short cases, objective structured clinical examination (OSCE), objective structured practical examination, portfolios and logbooks. The use of standardized patients in Saudi medical schools is minimal. External examiners from other Saudi medical schools as well as from other countries participate in the development and administration of these various examination processes.

A mandatory rotating internship year is required of all students before the medical diploma (either an MBBS or an MBChB) is awarded by a medical school. The medical diploma qualifies the graduate as a competent physician with the ability to practice medicine as a general practitioner anywhere in Saudi Arabia. If the graduate chooses to apply for a residency program in Saudi Arabia, he or she must sit for the "Acceptance Test", also known as Saudi Licensing Exam, regulated by the Saudi Commission for Health Specialties (SCHS) [20]. The "Acceptance Test" consists of 100 MCQs and lasts for 2.5 hours. In addition, some residency programs require specialty-specific exams, regulated by the SCHS, to screen candidates for entry into their programs. Established by a Royal decree in 1993, the SCHS is a scientific body with a corporate entity that has multiple roles focused mainly at the level of the postgraduate training programs and practicing healthcare professionals. These include the provision, supervision and accreditation of residency programs in the country in addition to the annual assessments and final certification examinations of residents in all healthcare specialties. SCHS also governs the registration process of all healthcare professionals, including the verification of credentials and the conduct of specialty-based equivalence examinations for foreign medical graduates with specialty certificates obtained abroad. Yet, SCHS has a limited role in supervising undergraduate medical education.

In addition to the establishment of new medical schools, the government has facilitated the sponsorship of many medical students to study abroad to face the increasing need for medical professionals. This has resulted in a large number of students studying medicine in countries like the United States, Canada, Australia, New Zealand, France, Germany, Holland, Ireland, Austria, Poland, Slovakia, Pakistan, Malaysia, and China, in addition to the Arab countries, Egypt, Jordan and Bahrain.

To summarize, we can identify six phenomena in the Saudi medical education movement: 1) the increase in the number of new medical schools over a relatively short period of time; 2) the wide spectrum of educational philosophies, instructional methods and assessment techniques; 3) the absence of published national agreement on the competency of medical graduates or standards for medical school graduation outcomes; 4) the large proportion of foreign medical graduates practicing medicine in Saudi Arabia; 5) the large number of Saudi medical students sent on scholarship to a wide spectrum of medical education systems around the world (from China in the Far East, to Slovakia in Eastern Europe, to Austria in Western Europe, to Canada in North America); and 6) the exponential increase (150% to 200%) in the enrollment of medical students in the established medical schools with no proportional increase in resources. In addition, there has been a heightened public awareness of the challenges facing the Saudi medical system as presented in the local media. These include the need for physician accountability, assurance of patients' safety and frequent reports on medical errors.

### Vision of the Saudi medical licensing examination

Acknowledging that the field of educational measurement and assessment is a science by itself, it is beyond the scope of this debate to discuss the psychometric characteristics of medical education examinations and tests. We will briefly outline the vision of a proposed Saudi Medical Licensing Examination hoping to trigger some discussion around the structure, objectives, timing and other details of the exam.

As stated above, deciding on what and how to test the students depends on the curriculum and the instructional methods used. Currently, there is no published consensus on the national competencies for medical education in Saudi Arabia. On the contrary, other countries have consensus of lists of competencies that a graduating medical student is expected to master in order to practice medicine, thus it would be easier to design national licensing examinations to address these different competencies. Table 2 summarizes examples of national and international competencies of physicians. Recently, there have been unpublished efforts by some educators and researchers to develop national medical education competencies in Saudi Arabia [21].

**Table 2:** Examples of National Competencies of Physicians

**Canada (CanMEDS)**[43]	**ACGME^1 ^Core Competencies**[44]	**GMC's^2 ^Good Medical Practice**[45]	**IIME GMER**^3^[46]
1. Medical expert	1. Patient care	1. Good clinical care	1. Professional Values, Attitudes, Behavior and Ethics
2. Communicator	2. Medical knowledge	2. Maintaining good medical practice	2. Scientific foundation of medicine
3. Collaborator	3. Practice-based learning and improvement	3. Relationships with patients	3. Communication skills
4. Manager	4. Interpersonal and communication skills	4. Working with colleagues	4. Clinical skills
5. Health advocate	5. Professionalism	5. Teaching and learning	5. Population health and health systems
6. Scholar	6. Systems-based practice	6. Probity	6. Management of information
7. Professional		7. Health	7. Critical thinking and research

^1^ACGME: Accreditation Council for Graduate Medical Education.

^2^GMC: General Medical Council.

^3 ^IIME GMER: Institute of International Medical Education Global Minimum Essential Requirements in Medical Education

#### Preparation of high-stakes examinations

As explained concisely by Roberts and colleagues [22], the four steps involved in preparing high-stakes assessment exams are: 1) blueprinting the educational objectives, 2) selection of appropriate test formats, 3) applying assessment strategies to achieve adequate levels of reliability, and 4) implementing appropriate standard setting and decision-making procedures.

Blueprinting or creating a table of specifications provides a grid which maps the content of the examination against the educational goals and learning objectives of the planned curriculum. Blueprinting ensures that the content and face validity of the test are established. The discussion of types of validity is beyond the scope of this debate [23].

**Figure 2:**
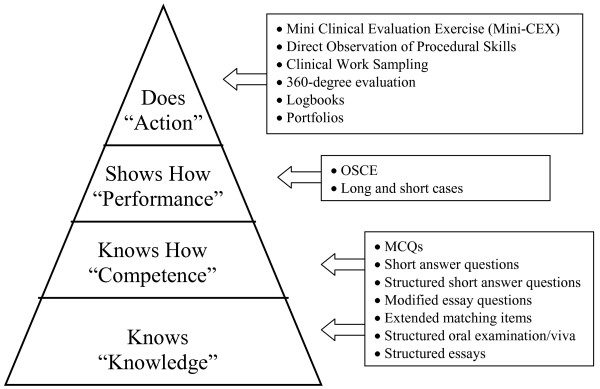
**Miller's Framework of Clinical Assessment **(^© ^Miller GE: The assessment of clinical skills/competence/performance. *Acad Med *1990, 65: S63–S67. Figure 1 [25]. Reproduced with the permission of the copyright holder.): with the corresponding appropriate methods of assessment.

The second step in the preparation of a high-stakes examination is the selection of test formats best suited to the educational objectives to be assessed. Initially, it is important to acknowledge that there is no one single test format that is able to assess all aspects of clinical competence [24]. From one perspective, it is important to decide on the level of assessment we intend to evaluate when assessing the clinical competencies as presented in the framework proposed by George Miller [25] (Figure 2). In particular, we need to decide on the knowledge, competencies, performances or actions of the medical students we are interested in measuring. Deciding on Miller's levels of assessment will help guide the choice of test format. Amin and colleagues described different methods of assessment, along with their strengths, weaknesses and the available evidence to support or refute its use [26]. Figure 2 summarizes the appropriate methods for each level of assessment according to Miller's framework.

In addition to the hierarchy of the assessment process, it is important to consider the three domains of learning competencies being measured: knowledge (cognitive), skills (psychomotor) and attitudes (affective). For example, if the examiner is interested in assessing whether the student knows the anatomy of the hip, this would be a cognitive domain question. If the examiner is interested in judging whether or not the student is able to perform a physical examination of the hip joint, this would be a psychomotor domain assessment. If the examiner is interested in assessing the student's communication and teamwork skills in a trauma situation, this could reflect an affective measure of the student's attitudes towards colleagues and other healthcare professionals. Although an MCQ or a short answer question test format can be used to answer the first question, an OSCE or observed short case would be a better format to address the second domain, and an OSCE with standardized patient or Mini-CEX is probably the best format to use to assess the third domain. Within each domain, there is taxonomy of levels of assessment, starting from simple basic tasks to advanced complex functions (Table 3). It is recommended that an examination consists of a balanced number of questions from different levels of the taxonomy within each domain.

**Table 3:** Taxonomy of Educational Objectives: Knowledge (Cognitive), Skills (Psychomotor) and Attitude (Affective) Domains

**Knowledge (Cognitive) **[28]	**Skills (Psychomotor) **[47]	**Attitudes (Affective) **[48]
**1. Knowledge: **recall or recognize information	**1. Perception: **uses sensory cues to guide actions	**1. Receiving: **demonstrates a willingness to participate in the activity

**2. Comprehension: **translate, interpret, extrapolate but not see full implications	**2. Set: **demonstrates a readiness to take action to perform the task	**2. Responding: **shows interest in the activity by seeking it out

**3. Application: **apply abstraction or general principles to concrete situations	**3. Guided response: **knows steps required to complete the task	**3. Valuing: **internalizes an appreciation for the activity

**4. Analysis: **separation of a complex idea into parts and understanding of relationship between the parts.	**4. Mechanism: **performs task in a somewhat confident, proficient, and habitual manner	**4. Organization: **begins to compare different values and resolves conflicts between them to form an internally consistent system of values

**5. Synthesis: **creative, mental construction of ideas from multiple sources to form complex ideas into a new integrated and meaningful pattern.	**5. Complex overt response: **performs task in a confident, proficient, and habitual manner	**5. Characterization by a value: **adopts a long-term value system that is "pervasive, consistent, and predictable".

**6. Evaluation: **to make a judgment of ideas using external evidence or self-selected criteria substantiated by observations or informed rationalizations.	**6. Adaptation: **performs task as above, but can modify actions to account for new situations	

	**7. Origination: **creates new tasks or objectives incorporating learned ones.	

The third step in preparing high-stakes examination is applying strategies to achieve acceptable reliability of the test. Reliability of a test is the extent to which the results can be reproduced on more than one occasion. In other words, it is a reflection of the consistency of the test. There are three common types of reliability for educational tests: intra-rater, inter-rater and test-retest reliability. We will briefly explain the concept of reliability, but suffice it to say that all testing will involve some error in the measurement process. This means that, regardless of how sophisticated and rigorous the examination methodology might be, the final test score (observed score) that the student achieves is actually the sum of his/her true score (which no one can measure) and sources of error measurement. The aim of all educational psychometricians is to minimize this error of measurement thus increasing the overall reliability of the testing format used to measure students' knowledge, skills and attitudes. There are a number of factors that can contribute to errors of measurement: variation in the difficulty of questions from one exam to another, variation of examiners' leniency and training on assessment protocols, variation in the complexity of the patient encounters, or variation in the examination procedure itself (e.g., use of paper and pencil versus computer, time of day, location). All of these potential sources of variation, which add noise or confounding factors that influence students' scores in testing, are detrimental to the overall reliability of the examination. The detrimental effects of these potential sources of variation can be remedied by using peer-reviewed blueprints (or rubric) of the exams and conducting educational workshops and meetings for the examiners to assure that the best practices of developing MCQs and OSCEs are followed.

The fourth step in preparing high-stakes examination is standard setting. It is the process which determines the borderline or minimum performance level between those students who pass the exam and are deemed acceptable from those who fail the exam and are deemed unacceptable. Although there are multiple methods for standard setting, they can be broadly classified as relative (norm-referenced) or absolute (criterion-referenced) [27]. As most summative and licensure examinations are considered to be high-stakes testing, the criterion-referenced standard setting method is preferred for both written and clinical performance exams. Standard setting requires the input of a number of expert judges who determine independently the criterion from which a minimally competent student must achieve in order to pass.

#### The proposed examination

**Figure 3:**
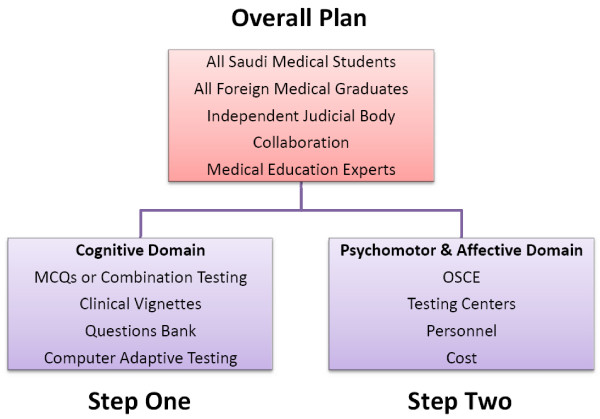
Summary of Proposal.

We are proposing an examination that consists of two parts (Figure 3). The first part tests the basic science and clinical knowledge and the second part tests the clinical skills and attitudes. We propose the two parts to be taken as a requirement for licensing medical doctors in Saudi Arabia. For example, for medical students, these should be taken during the last year of medical school. For foreign medical graduates, it would be a pre-requisite prior to any specialty-equivalence assessment they are required to perform.

The licensing examination should be conducted and maintained by an independent judicial body (for example, SCHS) with representation from the medical schools. Such an organization, especially at the initial stages of such a project, would likely benefit from consulting experienced organizations in conducting such examinations (e.g., the Medical Council of Canada and the National Board of Medical Examiners). Additionally, we suggest recruiting medical education experts in psychometrics and testing to assist in the development and administration of these examinations seeking higher test standards.

The first part of the medical licensing examination would be designed to test the cognitive domain of medical students' understanding of the main specialties with respect to the fundamental basic and clinical sciences knowledge. We specifically propose the use of 5 option one-best answer MCQs (A-type), or a combination of these MCQs with extended matching questions (R-type) and short answer questions. It is recommended that the questions are written to assess higher cognitive levels (application, analysis, synthesis and evaluation) of Bloom's taxonomy [28] (Table 3), than just recall or comprehension. More over, the questions should be based on clinical vignettes to increase the likelihood of integrating the clinical sciences (e.g., diagnosis, investigation and management options) and, thereby, increasing the clinical relevance of the exam. We invite interested readers to consult a very helpful resource published by the National Board of Medical Examiners for constructing effective MCQs [29]. We suggest considering the use of Item Response Theory in maintaining a Questions Bank to enable computer adaptive testing in the future. One of the advantages of computer adaptive testing is the potential to use fewer numbers of questions without endangering the overall reliability of the test and, simultaneously, avoiding the risk of depleting the items held within the Questions Bank too quickly.

The second part of the licensing examination would focus on the testing of psychomotor (skills) and affective (attitudes) domains of competencies (e.g., history taking, physical examination skills, communication skills, critical thinking, decision-making, breaking bad news, ethics, counseling skills, etc). We propose the use of an OSCE-style examination format that utilizes trained standardized patients and standardized checklists. Although the OSCE format has been found to have good reliability and validity when upwards of 16 stations are used, the expenses to develop and administer this type of examination process can be significant. In addition to having the appropriate facilities and personnel to maintain such a testing centre, there are costs related to the hiring and training of the standardized patients and examiners (raters) that are crucial to ensure the high reliability and validity of the results.

Given the comprehensiveness and standardization in the planning, writing and conduct of these rigorous examinations, these licensing examinations can eventually replace the multiple exams required currently from medical students which include the final year examinations, the SCHS' "Acceptance Test", and the specialty-specific screening exams.

### Driving forces for the national examination

#### 1. The increased number of medical schools

The exponential increase in the number of medical schools in Saudi Arabia in a short period of time necessitates a mechanism to ensure high-quality of graduating medical students both from the established and new medical schools. The established medical schools are at a disadvantage due to relocation of some of their experienced medical educators to the new schools, while the new medical schools are often disadvantaged due to being "new" in the business. Although a national medical licensing examination is not the only safeguard to ensure high quality of curriculum, instruction and learning outcomes, it is a tool with a tangible and standard measurable outcome that can be used to identify areas in need for potential improvement in the curriculum and instructional methods used in all medical schools. Although we do not have evidence of variation in the quality of graduates of Saudi medical schools, the need for a quality assurance exam cannot wait for the availability of such evidence. Evidence from other countries, however, is available. Data from the UK showed marked differences in candidates' scores on the examination for membership of the Royal College of General Practitioners when compared by medical school of qualifications [30,31]. In addition, despite their stringent accreditation system, education standards are different among medical schools [32,33] and significant variation in examination performances exists [34].

#### 2. Lack of consistency in assessment methods

The lack of consistency in using valid and reliable assessment methods across Saudi medical schools is an area of potential concern. Although we could not find published reports of the assessment methods used in Saudi medical schools, our personal communications and the fact that the authors of this report represent medical educators from many medical schools across the country confirm our position. We quote and echo Fowell et al.'s areas for concern based on the results of a survey of assessment practices of medical schools in the UK in 1998 [34]. The authors stated: "*Assessment methods and practices that might be considered to be less educationally desirable are still used by many schools, such as true/false multiple-choice questions (MCQs), negative-marking of MCQs, essays, patient management problems and oral assessments. There is an apparent lack of knowledge of technical aspects of assessment, particularly regarding the use of test 'blueprints' or 'matrices' in assessment design, robust approaches to standard-setting, and the use of item analysis as an approach to evaluation of assessment*".

#### 3. Large proportion of foreign medical graduates in Saudi Arabia

Foreign graduate physicians are present in large numbers in Saudi Arabia to meet the country's health service demands. The standard of their medical training and minimal competencies needs to be tested in a unified testing standard. Although there are examinations conducted by the Saudi Commission of Health Specialties for the purpose of "Professional Classification", these examinations are specialty-based and are not necessarily tailored to address the minimal competencies of a medical professional. On the contrary, in the US or Canada, any foreign medical graduate must pass licensing examinations in order to be eligible to practice in the country, then he or she should enroll in a residency program or under certain conditions, sit for the specialty-specific examination. Therefore, medical practitioners share a common licensing scheme which ensures every graduate has at least the minimum competence to function as a physician, according to the licensing body standards. Even in some countries where there is no medical licensing examination for national graduates, foreign medical graduates have to pass a licensing examination before they are able to practice medicine in these countries. Examples include the Professional and Linguistic Assessments Board (PLAB) Test in UK and the Australian Medical Council examination in Australia.

#### 4. Scholarships for undergraduate medical students

The expansion of international scholarship of undergraduate medical studies will bring many Saudi physicians with variable qualities of undergraduate medical education and from different cultural backgrounds into the country. The licensing exam will serve as a method of unifying the minimum standard of acceptable competency in the health service among these graduates.

#### 5. Planned, delivered, assessed and hidden curricula

**Figure 4:**
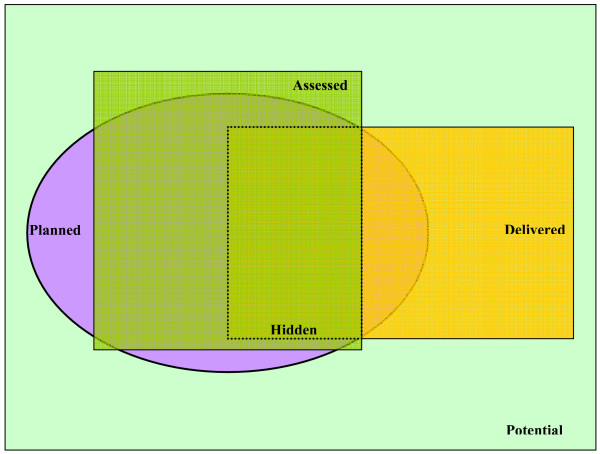
**Curricular Challenges** (^© ^Hays R. *Teaching and Learning in Clinical Settings*. Oxford: Radcliffe Publishing; 2006 [35]. Reproduced with the permission of the copyright holder.)

Though this article focuses on the assessment aspect of medical education, yet one cannot ignore its relation to curriculum, as it influences the curriculum both explicitly and implicitly. Richard Hays had nicely illustrated the differences and relationship between potential, planned, delivered, assessed and hidden curricula [35]. He defined the *potential *curriculum as one that reflects all of the possible content that curriculum planners could think of. The *planned *curriculum is what the planners have agreed to include in the curriculum for feasibility and relevance reasons. This will include long lists of educational goals and learning objectives. The *delivered *curriculum is the delivery of the planned curriculum, matching different instructional methods (lectures, small group discussions, clinical rotations) to address various learning objectives. There is a potential mismatch between the *planned *and *delivered *curriculum if the teachers are not aware of the details of the learning objectives of the planned curriculum. Hence, teachers may teach students most aspects of the planned curriculum, yet expand further to aspects of the potential curriculum at the expense of not covering other aspects of the planned curriculum. The *assessed *curriculum is what students learn because they expect it will be assessed. Students can get clues of what is important in the curriculum, by common sense, asking the help of their senior colleagues, and sensing the important aspects of the curriculum stressed on by the teachers. This is more likely to happen when the examinations are conducted at the local level. Ideally, the planned, delivered and assessed curriculum should overlap perfectly, but if any mismatch happens, an undesirable kind of curriculum emerges, called the *hidden *curriculum, which could potentially impede the achievement of the planned educational objectives. The concept of this curricular war is illustrated in Figure 4. We believe that a national examination will minimize this hidden curriculum because the national planned curriculum will be explicitly stated and the assessed curriculum will be similarly explicitly stated ensuring that most of the planned curriculum is assessed. Results of the examination will help determine whether medical schools are delivering the curriculum as planned or not.

#### 6. Completing the standardization process

Interestingly, the decisions to admit students into medical schools are based on the national standardized tests conducted by the National Center for Assessment in Higher Education. Similarly, residents are assessed for fitness to practice based on national standardized tests conducted by the Saudi Commission for Health Specialties. The standardization of the assessment of medical students prior to their entry into medical practice, whether they join residency or not, is needed to complete the first and last step of the standardization process already in practice.

### Hindering factors against the national examination

#### 1. Efforts and costs to maintain the process

As we explained earlier in our vision of this examination, this is a very arduous process. The planning, writing, revising, scoring and analyzing of these examinations are very complex and tedious processes that require the allocation of quite a significant amount of time, money and personnel. We believe that this is the strongest barrier against implementing such system. At the time of writing this manuscript, the fee for the Medical Council of Canada Qualifying Examination part I was around $700 US and for part II (a clinical examination) was $1,500 US, while the fee for Step 1 of the United States Medical Licensing Examination was $695 US, for Step 2 Clinical Knowledge was $695 US and for Step 2 Clinical Skills (a clinical examination) was $1,200 US. The expertise of the educators and researchers from the Medical Council of Canada, the National Board of Medical Examiners and the General Medical Council in conducting the standardized MCQ exams and OSCE patient encounter assessments would be beneficial to ease the implementation of these testing formats.

#### 2. Opposing arguments

The natural resistance to change is expected from both the medical schools' administrators and students. Interestingly, this was not the case in Khan and Sear's national online survey of 401 final year medical students' opinion of the General Medical Council's proposed reform of the undergraduate medical assessment system [36]. The majority (92.5%) of the respondents were in favor of consistency of assessment between medical schools. There was a nearly equal division in the number of students who were for and against the national exam to be used as a tool in the grading and ranking of students. Interestingly, 72.3% of the respondents were in favor of the exam being held near the end of the undergraduate course as a prerequisite for graduation and 45.4% of respondents thought that the national exam would lead to a fairer selection process for foundation jobs by allowing grading and marking.

There is an argument that the current SCHS' "Acceptance Test", also known as Saudi Licensing Exam, is enough and might perceive this proposal as redundant. Our rebuttal to this argument is three folds. First, we want to establish a mandatory test for every physician to practice medicine in Saudi Arabia, regardless of his school of graduation and regardless of whether he or she is interested to join a residency program or not. The licensing exam will ensure that minimal competencies are achieved by every practicing physician in Saudi Arabia. This is not the case in the current "Acceptance Test". It is utilized mainly as a screening tool for entry into residency programs in Saudi Arabia. Second, the proposed licensing exam will consist of a written part and OSCE part to ensure that competencies such as history taking, physical examination, and communication and counseling skills are achieved by practicing physicians with acceptable standards. This is not the case in the 100-MCQ "Acceptance Test" conducted by the SCHS. Third, the proposed mandatory licensing exam will function as a benchmark for medical schools to gauge their curriculum and instructional strategies. The SCHS' "Acceptance Test" is not suitable to provide this information for medical schools because only students who want to enroll in a Saudi residency program sit for this test.

#### 3. Exposing potential weakness in medical schools education system

Medical schools might resist this call for change fearing that they will be exposing the weaknesses of their medical schools publicly. This is definitely a valid fear as stakeholders from no one medical school would like to be singled out without an opportunity to address these concerns initially. However, the potential embarrassment can be avoided by keeping the results of the medical schools performance confidential for the first 5 years of implementing the system. This will give the poor performing medical schools time to rectify their problems. The licensing exam has the potential for helping medical schools identify the potential weaknesses in their education system early, which is of crucial importance for new medical schools. The licensing exam will enable them to identify and rectify problems as soon as they are detected.

#### 4. The fear of impeding flexibility within medical school's curriculum

Medical schools educators might resist the change because they believe that national learning outcomes and a national standardized examination will restrict the flexibility of customizing their curriculum and choice of assessment methods. Although there may be some truth in this, the advantage of ensuring that all curricula are meeting a minimal competency standard (national core curriculum) will allow for that flexibility to be maintained. Therefore, as long as the medical school has fulfilled the national curriculum, each is free to complement their respective curriculum with additional materials and resources.

#### 5. Language barrier for non-Arabic speaking physicians

English is the language of instruction and examination in all medical schools in Saudi Arabia. Implementing a mandatory OSCE part which entails communicating with patients, who are mostly Arabic-speaking, might be a major issue for non-Arabic speaking physicians. Although these physicians might be very competent medically, they might have difficulty communicating in Arabic, at least initially upon their arrival to Saudi Arabia. The solution to this problem might be the introduction of basic Arabic courses for physicians.

### Strengths of the national medical licensing examination

First, the use of a standardized national exam will ensure that any medical graduate or practicing doctor in Saudi Arabia has achieved at least a common standard of medical knowledge and clinical skills competencies. This does not necessarily mean that all graduates are homogenous. It will ensure that minimal standards are met and will maintain the high reputation of Saudi Arabia-trained physicians.

Second, this exam and the process of assessing both knowledge as well as clinical and generic skills will improve the public trust and confidence in the Healthcare in Saudi Arabia. It will ensure that physicians are competent in their clinical skills and attitudes in addition to their competence in medical knowledge.

Third, the use of results of a national examination is a reliable, transparent and valid measure of candidates' qualifications for jobs and residency programs' application. It will avoid stereotyping students graduating from lenient medical schools as lower performers thus providing equal and fair opportunities in the current competitive job market.

Fourth, the exam can be used as a tool for quality assurance to benchmark medical schools across Saudi Arabia, and to correct any potential problem or deficiency in the curricula or methods of instruction at any medical school.

Fifth, although we do not necessarily propose the exclusive use of this examination as a method of assessing students for readiness to practice, since there are other aspects of competencies (such as professionalism) that cannot be assessed thoroughly by such an examination. The implementation of a standardized national exam (which potentially substitutes for the final exams in medical schools) will decrease the time constrains on the faculty (teachers and educators) thus allowing more time for ongoing global assessment of the learners and the exploration of new methods to rectify the identified weaknesses in medical curricula.

Sixth, if this examination is eventually implemented and proved to be successful in measuring standards of students' performance, Saudi Arabia can become the regional center for the administration of high quality medical licensing examinations that would address the needs of other Arab countries interested in moving towards this process.

### Weaknesses of the national medical licensing examination

First, although we discussed standardization of assessment as a strength, this is considered by some as a weakness [37]. Their rationale is that standardization of assessment will force medical schools to eventually standardize their curriculum and instructional methods. Hence, this will eliminate the creativity and innovation in instructional methods. However, this did not happen in Canada, where national licensing examination has been in place for years. On the contrary, in the face of a common national examination for all Canadian medical schools graduates, the problem-based learning approach was initiated at McMaster University. The rationale of opponents of a standardized national examination is that it focuses on the outcome rather than the process of medical education. Some are concerned that both students and staff of medical schools will be exam-oriented. Their focus will be on how to pass the exam and keep the reputation of the school high, and less on how to graduate students as competent physicians. We disagree with these concerns since there is enough evidence that testing improves the learning process and that testing yields better long term retention than repeated studying [38].

Second, there is a concern that the implementation of a national examination will stratify medical schools into explicit league tables, which could be detrimental for morale of staff and students in medical schools with poor performance. This is a true concern. However, it does not justify avoiding a national examination. On the contrary, if such poor performing medical schools are identified based on the results of the national examination, measures can be taken to rectify the problems early, thus preventing the problem from worsening. We believe that, even if there is not a national examination, there will be implicit league tables of medical schools, which could be detrimental for graduating students from the implicitly poor performing schools. Regardless of how genius, hard working, and innovative the students are, they will be perceived as graduating from a lenient medical school. This will add noise and confusion to the decision of their admission to residency programs and job applications. However, if a standardized national examination exists, their performance on the examination will be easily compared against that of their colleagues from other schools regardless of how lenient or strict their medical school is perceived as being.

Third, some authors argue that a one time assessment is not as comprehensive as ongoing assessments of medical schools [37]. We totally agree with this opinion. We are not proposing an exclusive national exam while discounting other important or relevant methods of assessment used by individual medical schools. Instead, we propose the use of the national exam to ensure that minimal competencies are met for all graduating medical students. This can be supplemented by any number of other methods of ongoing assessments used by the medical schools.

Fourth, this test will definitely burden the students with additional financial commitments. However, students in the government-funded medical schools in Saudi Arabia are already at an advantage in that they can study medicine for free with additional financial support available from the government. As professionals in the discipline, the very least we can do is to make a commitment that we meet recognized national standards for the practice of medicine in Saudi Arabia. Furthermore, we anticipate that the government might be supportive in moving towards this process by subsidizing parts of the exam to lessen the burden on the students.

### Alternatives to national medical licensing examination

#### Development of a body of accreditation

Although there is no formal undergraduate medical accreditation organization in Saudi Arabia, the recent establishment of the National Commission for Academic Accrediation and Assessment [39] is promising. The commission has recently developed an accreditation system of medical schools in Saudi Arabia; the report is yet to be published. In order for any accreditation body to maintain the highest standards of medical education, its policies and decisions should be transparent and public and it should have enough administrative power to enforce needed changes in any medical school. Furthermore, accreditation is not an alternative to a national licensing exam. Both are complementary and help in competency assessment of our medical graduates.

#### External examiners

Medical schools in most countries, including Saudi Arabia, use external examiners (from outside the medical school) to ensure that national and international standards are met. Forbes discussed, in an overview, the requirements and duties of external examiners [40]. External examining is not just the mere visit of another medical school during the final examination period. Karuhije and Ruff [41] identified six steps in the external examining experience: 1) appointment, 2) contract, 3) review of curriculum and examination materials, 4) on-site visit, 5) consolidating internal and external assessment, and 6) preliminary and final reports. Sheehan [42] identified 15 facets for the role of external examiner: subject expert, experienced, impartial judge, custodian of standards, interpreter of regulations, rapid reader, board member, decision maker, conflict coper, interviewer, power source, signatory, migrant, reporter and outsider. Apart from the difficulty of finding persons with the aforementioned characteristics, the process of external examining is expensive, including the remuneration, travel, accommodation and hospitality costs. Considering there are 25 medical schools, the cost of multiple external examiners for each medical school can be substantial, yet the outcomes of external examining are not as objective as a national licensing examination.

#### Establishment of a national center for the assessment of medical competencies

As explained earlier, the process of designing, refining, running and scoring methodologically-sound high-stakes examinations is complex and time consuming. Hence, an alternative to a national medical licensing examination is the establishment of a national center for assessing medical competencies. The function of such a center will be to provide training, advice and technical support in the planning, conduct and analysis of different methods of medical education assessment conducted by local medical schools. Additionally, the center may host a shared bank of assessment methods that can be used by any medical school in the region. Yet, security of the shared bank of assessment methods is an issue that needs to be addressed.

## Conclusion

The establishment of a large number of new medical schools in Saudi Arabia opens the doors for many medical schools graduates to satisfy the country's health care needs. However, it calls for a quality assurance system to guarantee that acceptable international standards of medical competencies are met by our medical graduates. We view the need for the national licensing medical examination as a necessity, as it is timely-critical for the best of our health care system. Table 4 summarizes the pertinent points in our debate. We hope this proposal will trigger constructive discussions among Saudi medical schools, educators, teachers and students to reach a consensus for the best of our country.

**Table 4:** Summary of Driving and Hindering Forces along with strengths and weaknesses of a national medical licensing examination in Saudi Arabia

**Driving Forces**	**Hindering Forces**
1. Increase in number of medical schools over short period	1. Cost and time
2. Inconsistency and variable expertise in using valid and reliable assessment methods	2. Natural resistance to change
3. Large number of foreign medical graduates	3. Fear of discovering medical schools' weaknesses
4. Saudi medical students on scholarships to various countries	4. Restriction of medical schools freedom and flexibility on the choice of curriculum and assessment methods
5. What is taught is not necessarily what is learnt. Issues of planned, delivered, assessed and hidden curriculum	5. Difficulty on agreement on a set of educational objectives
6. Standardized testing for admission to medical schools and exit from residency calls for standardization of exit from medical school	6. Language barrier for non-Arabic speaking physicians in the OSCE part of the licensing exam

**Strengths**	**Weaknesses**

1. Standardization of medical education leads to graduating medical students with the minimal required competences	1. Standardization of medical education leads to loss of creativity and innovation required of a critical thinker physician
2. Strengthens public trust and maintains the reputation of Saudi trained physicians	2. League tables might be potentially detrimental to the morale of staff and students of "weak" medical schools
3. Fair assessment of medical students and selection of candidates into residency program and jobs pool	3. The risk that students might be exam-oriented
4. Provides quality assurance and feedback on curriculum implementation and instructional methods across all medical schools	4. One time assessment is not as comprehensive as ongoing assessments of medical schools
5. Frees medical teachers to teach and do research and leaves the complexity of conducting examinations to the national organization	5. Burdens the students with additional financial commitment
6. Saudi Arabia may act as a regional center for high quality medical licensing examination	

## Competing interests

The authors declare that they have no competing interests.

## Authors' contributions

SB conceptualized the idea and drafted the first manuscript. RZ initiated and facilitated the collaboration between co-authors. RZ and WA helped with data acquisition. TD provided critical supervision during the writing of the manuscript. All authors participated in the writing of and made essential contributions to this paper and critically reviewed and approved the final manuscript.

## Pre-publication history

The pre-publication history for this paper can be accessed here:



## References

[B1] Ness D (1990). Changes to the MCC's qualifying examination. CMAJ.

[B2] Hill MD (1992). Why another examination?. CMAJ.

[B3] (1992). Position statement. Licensure, postgraduate training and the Qualifying Examination. Federation of Medical Licensing Authorities of Canada, Association of Canadian Medical Colleges and Medical Council of Canada. CMAJ.

[B4] Kenyon A (1994). The Part II examination: more thoughts. CMAJ.

[B5] Kennedy B (1995). The Part II examination: political exercise or national standard?. CMAJ.

[B6] Hallock JA, Melnick DE, Thompson JN (2006). The step 2 clinical skills examination. JAMA.

[B7] Mehta NP, Kramer DB (2005). A Critique of the USMLE clinical skills examination. MedGenMed.

[B8] Barrett A (2005). National clinical assessments. StudentBMJ.

[B9] Koczwara B, Tattersall MH, Barton MB, Coventry BJ, Dewar JM, Millar JL (2005). Achieving equal standards in medical student education: is a national exit examination the answer?. Med J Aust.

[B10] McNeil HP, Grimm MC (2005). Achieving equal standards in medical student education: is a national exit examination the answer?. Med J Aust.

[B11] Cox K (2005). Achieving equal standards in medical student education: is a national exit examination the answer?. Med J Aust.

[B12] Fletcher K (2005). Nationally assessed. StudentBMJ.

[B13] Wass V (2005). Ensuring medical students are "fit for purpose". BMJ.

[B14] Patel K (2001). USMLE-style exam. Med Educ.

[B15] General Medical Council Education Committee (2006). Final Report: Strategic Options for Undergraduate Medical Education. Consultation.

[B16] Mufti MH (2000). Healthcare development strategies in the Kingdom of Saudi Arabia.

[B17] (2008). National Center for Assessment in Higher Education, Saudi Arabia. http://www.qiyas.org.

[B18] Alshehri MY (2001). Medical curriculum in saudi medical colleges: current and future perspectives. Ann Saudi Med.

[B19] Khalid BA (2008). The current status of medical education in the Gulf Cooperation Council countries. Ann Saudi Med.

[B20] (2008). Saudi Commission for Health Specialties. http://scfhs.org.sa/.

[B21] Zaini R (2008). Saudi Future Doctors.

[B22] Roberts C, Newble D, Jolly B, Reed M, Hampton K (2006). Assuring the quality of high-stakes undergraduate assessments of clinical competence. Med Teach.

[B23] Hays R (2006). Teaching and learning in clinical settings.

[B24] Wass V, McGibbon D, Vleuten C Van der (2001). Composite undergraduate clinical examinations: how should the components be combined to maximize reliability?. Med Educ.

[B25] Miller GE (1990). The assessment of clinical skills/competence/performance. Acad Med.

[B26] Amin Z, Chong YS, Khoo HE (2006). Practical guide to medical student assessment.

[B27] Norcini JJ (2003). Setting standards on educational tests. Med Educ.

[B28] Bloom BS (1956). Taxonomy of educational objectives: the classification of educational goals Handbook 1: Cognitive domain.

[B29] National Board of Medical Examiners. Constructing Written Test Questions For the Basic and Clinical Sciences. http://www.nbme.org/PDF/ItemWriting_2003/2003IWGwhole.pdf.

[B30] Wakeford R, Foulkes J, McManus C, Southgate L (1993). MRCGP pass rate by medical school and region of postgraduate training. Royal College of General Practitioners. BMJ.

[B31] McManus IC, Elder AT, de Champlain A, Dacre JE, Mollon J, Chis L (2008). Graduates of different UK medical schools show substantial differences in performance on MRCP(UK) Part 1, Part 2 and PACES examinations. BMC Med.

[B32] Boursicot KA, Roberts TE, Pell G (2006). Standard setting for clinical competence at graduation from medical school: a comparison of passing scores across five medical schools. Adv Health Sci Educ Theory Pract.

[B33] Boursicot KA, Roberts TE, Pell G (2007). Using borderline methods to compare passing standards for OSCEs at graduation across three medical schools. Med Educ.

[B34] Fowell SL, Maudsley G, Maguire P, Leinster SJ, Bligh J (2000). Student assessment in undergraduate medical education in the United Kingdom, 1998. Med Educ.

[B35] Hays R (2006). What can be learnt in clinical settings?. Teaching and learning in clinical settings.

[B36] Khan KZ, Sear JW (2007). A national online survey of final year medical students' opinion on the General Medical Council's proposed reforms to the undergraduate medical assessment system. Postgrad Med J.

[B37] Schuwirth L (2007). The need for national licensing examinations. Med Educ.

[B38] Eva KW (2007). Putting the cart before the horse: testing to improve learning. BMJ.

[B39] National Commission for Academic Accrediation and Assessment, Saudi Arabia. http://www.ncaaa.org.sa/.

[B40] Forbes CD, Dent JA, Harden RM (2005). External examiners. A Practical Guide for Medical Teachers.

[B41] Karuhije HF, Ruff CC (2002). External examiners: international collaboration in nursing education. ABNF J.

[B42] Sheehan J (1994). External examiners: roles and issues. J Adv Nurs.

[B43] The Royal College of Physicians and Surgeons of Canada. The CanMEDS Physician Competency Framework. http://rcpsc.medical.org/canmeds/index.php.

[B44] Accreditation Council for Graduate Medical Education. ACGME General Competencies. http://www.acgme.org/outcome/comp/GeneralCompetenciesStandards21307.pdf.

[B45] General Medical Council. Tomorrow's Doctors. http://www.gmc-uk.org/education/undergraduate/GMC_tomorrows_doctors.pdf.

[B46] Schwarz MR, Wojtczak A (2002). Global minimum essential requirements: a road towards competence-oriented medical education. Med Teach.

[B47] Simpson E (1972). The Classification of Educational Objectives, Psychomotor Domain.

[B48] Krathwohl DR, Bloom BS, Masia BB (1964). Taxonomy of educational objectives: the classification of educational goals Handbook 2: Affective domain.

